# Quercetin Suppresses AOM/DSS-Induced Colon Carcinogenesis through Its Anti-Inflammation Effects in Mice

**DOI:** 10.1155/2020/9242601

**Published:** 2020-05-21

**Authors:** Rui Lin, Meiyu Piao, Yan Song, Chunyan Liu

**Affiliations:** ^1^Department of Gastroenterology and Hepatology, General Hospital of Tianjin Medical University, Tianjin, China; ^2^The Department of Hematology, General Hospital of Tianjin Medical University, Tianjin, China

## Abstract

Colorectal cancer (CRC) is the fourth leading cause of tumor-related deaths worldwide. In this study, we explored the in vivo effects of quercetin, a plant flavonol from the flavonoid group of polyphenols with antioxidant effects, on colon carcinogenesis induced by azoxymethane/dextran sodium sulfate (AOM/DSS). Thirty mice were randomly assigned into three groups: the control group, the AOM/DSS group, and the quercetin+AOM/DSS group. CRC was induced by AOM injection and a solution of 2% DSS in the drinking water. In the AOM/DSS-induced colon cancer mice model, quercetin treatment dramatically reduced the number and size of colon tumors. In addition, quercetin significantly restored the leukocyte counts by decreasing the inflammation caused by AOM/DSS. We also observed that the expression of oxidative stress markers, such as lipid peroxide (LPO), nitric oxide (NO), superoxide dismutase (SOD), glucose-6-phosphate (G6PD), and glutathione (GSH), could be reduced by quercetin, suggesting that the anti-inflammatory function of quercetin comes from its antioxidant effect. Moreover, potential biomarkers were identified with serum metabolite profiling. Increased levels of 2-hydroxybutyrate, 2-aminobutyrate, and 2-oxobutyrate and decreased levels of gentian violet, indole-3-methyl acetate, N-acetyl-5-hydroxytryptamine, indoxyl sulfate, and indoxyl were also found in the AOM/DSS-treated mice. However, quercetin treatment successfully decreased the levels of 2-hydroxybutyrate, 2-aminobutyrate, 2-oxobutyrate, endocannabinoids, and sphinganine and increased the levels of gentian violet, N-acetyl-5-hydroxytryptamine, indoxyl sulfate, and indoxyl. Together, our data demonstrated that quercetin could maintain relatively potent antitumor activities against colorectal cancer in vivo through its anti-inflammation effect.

## 1. Introduction

Colon carcinoma is one of the most common malignant tumors observed in clinical practice [1]. Colorectal cancer (CRC) affects more than 1 million people each year worldwide [2]. In spite of improvements in early screening and treatment, colon carcinoma remains the fourth leading cause of tumor-related deaths in the world [3]. Due to the metastasis of tumor cells, over 30% of patients with CRC die within five years of their initial diagnosis [3]. Emerging evidence confirms that there is a marked survival difference among CRC patients that is largely due to the tumor stage at the time of diagnosis. For this reason, effective treatments for CRC are still needed.

A multistep process is involved in the transformation of normal colon epithelial cells to malignant cells. However, only a very small number of human CRC cells are genetically susceptible to the related disorders, such as juvenile polyposis, phosphatase and tensin homolog (PTEN) hamartoma tumor, hamartomatous polyposis syndrome, and others [4]. The majority of human CRC cases are caused by environmental risk factors rather than heritable genetic changes, including chronic intestinal inflammation, food-borne mutagens, and specific intestinal commensals [5]. Among these environmental risk factors, chronic inflammation is the most significant risk factor for CRC carcinogenesis. In addition, patients diagnosed with inflammatory bowel diseases (IBD), such as ulcerative colitis (UC) and Crohn's disease (CD), have a significantly higher risk of developing colitis-associated CRC (CAC) and have a higher mortality rate when compared to other CRC patients [6]. Furthermore, tumor-associated inflammation has been found in patient tissue samples from a large population of patients who did not show any signs of IBD prior to the initial stage of CRC and demonstrated its potential to promote cancer development in the gut, indicating the extremely important role of inflammation in CRC development [7].

A number of experimental mouse models of CRC have been generated by pioneers in this field, providing important insights into the pathogenesis mechanisms, drug discoveries, and validation of novel therapeutic targets for CRC [8, 9]. The AOM/DSS-induced mouse model of CAC is one of the most widely used chemically induced CRC models due to its high reproducibility and potency. Studies based on this model have demonstrated the importance of the inflammation process in CRC development and have elucidated some of the mechanisms of inflammation-related colon carcinogenesis in the gut, with an emphasis on the function of pro- and anti-inflammatory cytokines [8, 10].

Quercetin, a bioactive flavonol from the flavonoid group of polyphenols with antioxidant effects, is widely available in various edible plants [11–14]. Recently, studies have shown that quercetin increased intracellular reactive oxygen species (ROS) in colon cancer cells [15]. Moreover, the anti-inflammation ability of quercetin has been explored in other types of tumors and diseases [16–19]. However, some studies indicated that quercetin supplementation may have a role in accelerating colon tumor formation [20]. Therefore, whether or not quercetin can suppress the proliferation characteristics of colon cancer cells needs further investigation.

For these reasons, in this study, we explored the in vivo effects of quercetin on AOM/DSS-induced colon carcinogenesis. In addition, we evaluated the colon carcinogenesis, oxidative stress, and potential serum biomarkers in different groups of mice.

## 2. Materials and Methods

### 2.1. Animals

All animal experiments followed the guidelines for ethical procedures and scientific care given by the Animal Care and Use Committee of the General Hospital of Tianjin Medical University. Wild-type C57BL/6J mice were purchased from The Jackson Laboratory. All animals were housed in plastic cages under a 12 h light/dark cycle with free access to water and food. AOM (cat. no. 25843-45-2) was purchased from Sigma-Aldrich. DSS (cat. no. 160110) was purchased from MP Biomedicals, LLC (Aurora, OH, USA). The mice were randomly divided into two experimental groups (AOM/DSS and quercetin+AOM/DSS) and one control group (*n* = 10 per group). The mice in the AOM/DSS and quercetin+AOM/DSS groups were given a single intraperitoneal injection of AOM (10 mg/kg body weight). Seven days after the AOM injection, the mice were given 2% DSS (*w*/*v*) in their drinking water for 7 days. In addition, the mice in the quercetin+AOM/DSS group were given 30 mg/kg of quercetin in their diets throughout the experiment. All mice were sacrificed by cervical dislocation 4 weeks after the administration of quercetin.

### 2.2. Sample Collection and Preparation

All mice were anesthetized with ether and sacrificed by cervical dislocation. Blood samples were collected and kept at -80°C until hematological analysis. The colon of each mouse was isolated and fixed in 10% neutral buffered formalin for subsequent histopathological study. The tumor volumes and weight were measured at the end of the experiment. The rest of the parts were frozen with liquid nitrogen, then stored at -20°C for subsequent biochemical studies.

### 2.3. Hematological Parameter Determination

The red blood cell (RBC), white blood cell (WBC), lymphocyte (LYMP), and eosinophil counts were measured on an Auto Hematology Analyzer (Mindray BC-3200).

### 2.4. Analytical Measurements

The total protein content in the collected colon tissues was determined by bicinchoninic acid assay (BCA assay) (ab102536, Abcam). Lipid peroxide (LPO) levels were analyzed using a lipid hydroperoxide assay kit (ab133085; Abcam, Cambridge, UK) following the manufacturer's standard procedures. Nitric oxide (NO) levels were analyzed using a nitric oxide assay kit (ab65328; Abcam, Cambridge, UK) according to the manufacturer's standard procedures. Superoxide dismutase (SOD) activity was assayed using a kit (Cayman Chemical Co., Ann Arbor, MI, USA) following the manufacturer's standard procedures. Catalase activity (CAT) was determined using a catalase assay kit (707002; Cayman Chemical Co.) according to the manufacturer's standard procedures. The activity of glucose-6-phosphate dehydrogenase (G6PD) was determined using a glucose-6-phosphate dehydrogenase assay kit (ab102529; Abcam, Cambridge, UK) following the manufacturer's standard procedures. The activity of glutathione (GSH) was measured by using a commercially available assay kit (ab138881; Abcam, Cambridge, UK) according to the manufacturer's standard procedures.

### 2.5. Colon Histology

To prepare the specimens for microscopic imaging, colon tissue samples were processed in a series of increasing alcohols and xylene. Next, these colon tissues were embedded in paraffin. From the central segment, sections with a thickness of 8 *μ*m were obtained with a rotation microtome (Leica RM 2145, Wetzlar, Germany), then mounted on Superfrost Plus slides (Thermo Fisher Scientific, Waltham, MA, USA). Before staining, the colon sections were subjected to dewaxing and rehydration by immersing the slides consecutively in xylene and ethanol solutions. Hematoxylin and eosin (H&E) stains were used on these sections with standard methods and analyzed for the severity of colonic inflammation. Next, the severity of the areas of epithelial degeneration, focal or multifocal areas of consolidation, erosions of the epithelium, presence of ulcers, tissue hyperplasia, and size of the affected areas were assessed. The inflammatory index was then scored. The basal level of inflammation in the control group was scored from 0 to 3, a moderate increase over the control level of inflammatory cells was scored from 3 to 6, and a significant elevation was scored from 6 to 10 [21].

### 2.6. BrdU Staining

BrdU crypt cell labeling was used for the crypt cell proliferation measurement. For BrdU staining, colon tissue sections were first deparaffinized, then treated with proteinase K (Sigma-Aldrich, MO, USA). The tissue samples were then incubated overnight with an anti-BrdU antibody (clone B44, BD Biosciences), after which a secondary antibody (Pierce, Rockford, IL, USA) was added for 2 h. These sections were treated with a Vectastain ABC kit reagent (Vector Laboratories Inc.) for 20 min at room temperature. The color was developed using 3,3′-diaminobenzidine (Vector Laboratories Inc., Burlingame, CA, USA). Slides were then dehydrated, cover-slipped, and imaged.

### 2.7. Serum Metabolomics

A 100 *μ*L supernatant was combined with 200 *μ*L of methanol and shaken strongly for 60 s. Prior to the LC/TOF-MS analysis, centrifugation occurred at 12,000 rpm for a 10 min period under a temperature condition of 4°C. Random coding of every sample took place, and each sample was involved in a LC/TOF-MS analysis. To conduct the LC-MS analysis, a Shimadzu LC-30A system equipped with an ACQUITY UPLC® HSS T3 Column (150 mm × 2.1 mm, 1.8 *μ*m, Waters Corp., Milford, USA) was employed. This column was maintained at a temperature of 40°C. For the analysis of serum samples, the abovementioned column was used to facilitate the separation, and the temperature condition was again maintained at 40°C.

During the mobile phase, the constituents were (A) water with 0.1% formic acid and (B) acetonitrile. Several linear gradient conditions were used: 0–0.5 min, 2% B; 0.5–9 min, 2%–50% B; 9–12 min, 50%–98% B; 12–13 min, 98% B; 13–14 min, 98%−2% B; and 14−15 min, 2% B. The flow rate was 0.3 mL·min^−1^, and the injection of every sample was 5 *μ*L. The AB 5600+ mass spectrometer was used to conduct the ESI-MSn experiments, and the spray voltage was 5.50 kV in the positive mode and -4.50 kV in the negative mode. The curtain gas was set at 35 psi, while gas 1 and gas 2 were 50 psi. The source temperature was 500°C. A mass range of *m*/*z* 100–1,500 was given a full scan by the mass scanner, and a collision energy level of 45 eV was employed. A dynamic exclusion was applied, and the original LC-MS data were compiled into the mzXML format using ProteoWizard (v.3.0.8789). The processing was conducted with XCMS (v.3.3.2). Following the analysis of the data based on peak area normalization, we used SIMCA-P+ 13.0 (Umeå, Sweden) to apply a multivariate statistical analysis on the data matrix. To facilitate the visualization of the general separation, an unsupervised principal component analysis (PCA) was initially applied for every sample. Then, to gain insight into the differences across the various groups, a supervised partial least-squares discriminant analysis (PLS-DA) was used.

The detection and identification of metabolites took place using accurate mass and MS/MS information obtained from several electronic databases, including HMDB (https://www.hmdb.ca), METLIN (https://metlin.scripps.edu), MassBank (http://www.massbank.jp), LIPID MAPS (http://www.lipidmaps.org), and mzCloud (https://www.mzcloud.org). The MS/MS metabolite spectra were matched with structural information gathered from the electronic databases. Confirmation of the metabolites was undertaken based on a comparative analysis of retention times and fragmentation patterns against the standards.

### 2.8. Statistical Analysis

All statistical analyses were performed using GraphPad 7.00 (Prism) software. All results are expressed as mean values ± SD. A *P* value < 0.05 was considered statistically significant. Distinct and clear statistical results were commonly abbreviated with stars (e.g., ^∗^*P* < 0.05, ^∗∗^*P* < 0.01, and ^∗∗∗^*P* < 0.001). Student's *t*-test was used to compare the two groups. A one-way ANOVA with Bonferroni's posttest was used in comparisons of more than two groups.

## 3. Results

### 3.1. General Observations

**Figure 1:**
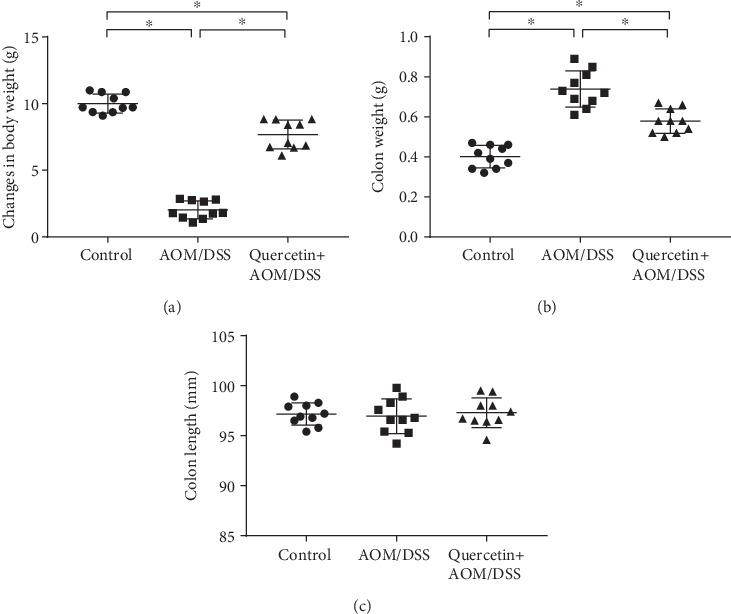
Quercetin restores body and colon weight in AOM/DSS-treated mice. (a) Quantification of body weight of control and experimental mice. (b) Quantification of colon weight of control and experimental mice. (c) Quantification of colon length of control and experimental mice. Data are expressed as mean values ± SD (*n* = 10/group). ^∗^*P* < 0.05, Student's *t*-test.

**Figure 2:**
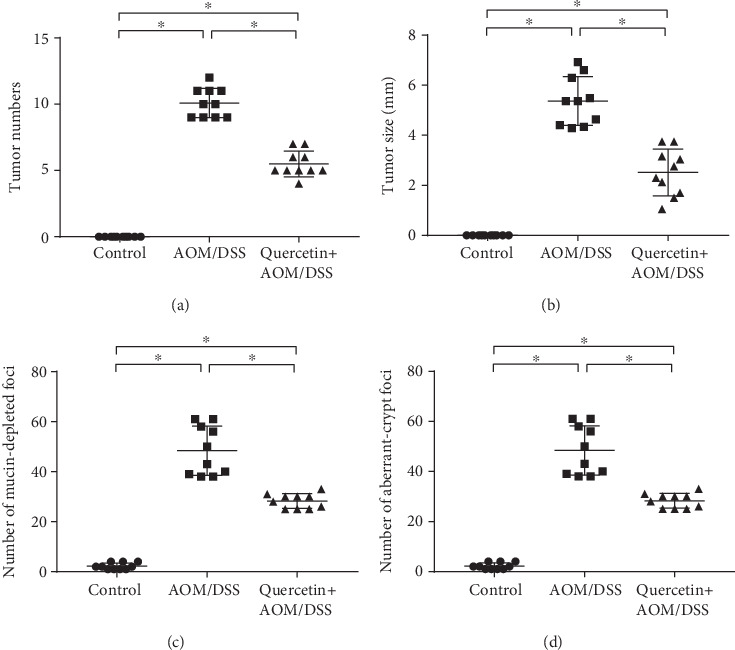
Quercetin reduces tumor incidence in AOM/DSS-treated mice. (a) Number of colon tumors in the control (*n* = 10), AOM/DSS-treated (*n* = 10), and quercetin+AOM/DSS cotreated (*n* = 10) groups. (b) Size of colon tumors in the control (*n* = 10), AOM/DSS-treated (*n* = 10), and quercetin+AOM/DSS cotreated (*n* = 10) groups. (c) Quantification of mucin-depleted foci in control and experimental mice. (d) Quantification of aberrant crypt foci in control and experimental mice. Data are expressed as mean values ± SD (*n* = 10/group). ^∗^*P* < 0.05, Student's *t*-test.

As shown in our study, mice receiving AOM/DSS in their drinking water exhibited a significant loss in body weight when compared to the control group (Figure 1(a)). Furthermore, the mean length of the colon of the mice treated with AOM/DSS was significantly longer than that of the control mice (Figure 1(b)). Of note, quercetin dramatically reversed the body weight and colon weight changes induced by the AOM/DSS in the mice (Figures 1(a) and 1(b)). Feeding mice with AOM/DSS alone or AOM/DSS together with quercetin did not produce any statistically significant colon length changes when compared to the control mice (Figure 2). These observations indicated that quercetin could reverse AOM/DSS-induced colon carcinogenesis in mice.

### 3.2. Quercetin Helps Protect the Hemopoietic System

**Figure 3:**
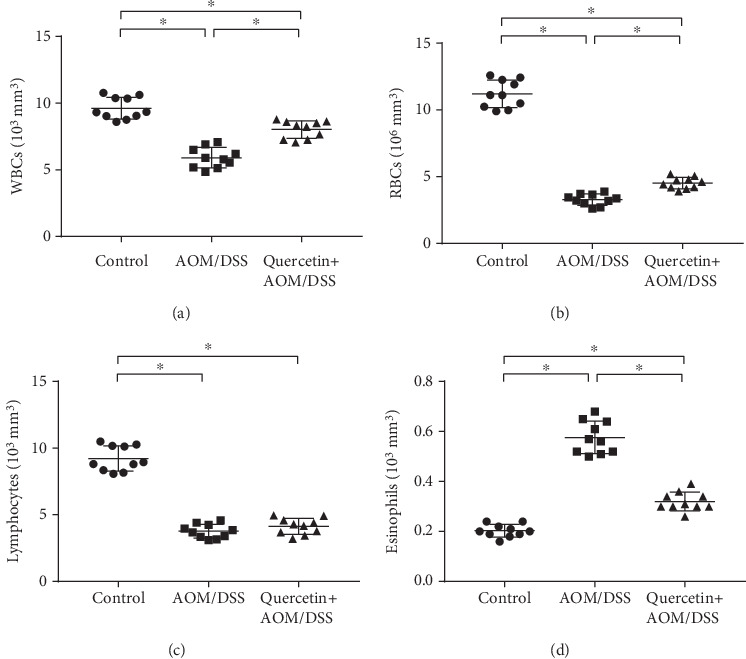
Quercetin helps protect the hemopoietic system. (a) Quantification of plasma concentration of WBCs in control and experimental mice. (b) Quantification of plasma concentration of RBCs in control and experimental mice. (c) Quantification of plasma concentration of lymphocytes in control and experimental mice. (d) Quantification of plasma concentration of eosinophils in control and experimental mice. Data are expressed as mean values ± SD (*n* = 10/group). ^∗^*P* < 0.05, Student's *t*-test.

Since quercetin is involved in body inflammation and immunity regulation [22], we investigated the alterations to the mice hematological parameters. After treatment with AOM/DSS, the mice exhibited significantly decreased leukocyte counts, including WBCs, RBCs, lymphocytes, and eosinophils (Figures 3(a)–3(d)). However, quercetin brought back the RBC, WBC, lymphocyte, and eosinophil counts more or less to near normal levels in the AOM/DSS-treated mice (Figures 3(a)–3(d)). This finding indicates that quercetin helps protect the hemopoietic system.

### 3.3. Quercetin Reduces Tumor Incidence and Inflammation in AOM/DSS-Treated Mice

**Figure 4:**
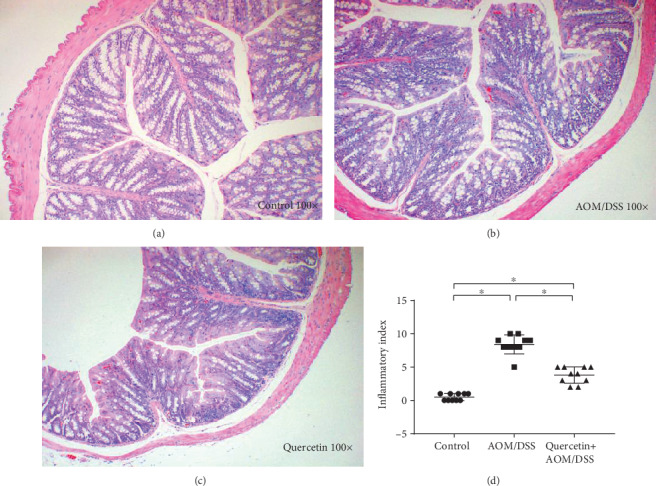
Quercetin reduces inflammation in AOM/DSS-treated mice. (a) H&E staining figure in the control group. (b) H&E staining figure in the AOM/DSS group. (c) H&E staining figure in the quercetin+AOM/DSS group. (d) Inflammatory index of the three groups. ^∗^*P* < 0.05, Student's *t*-test, data representative of three independent experiments presented as mean values ± SD (*n* = 10/group).

Next, we investigated the colon carcinogenesis in these mice by calculating the tumor number and weight. As shown in Figure 2(a), the mean number of tumors was 0 (*n* = 10) in the control group and 10 ± 1.04 (*n* = 10) in the AOM/DSS treatment group, while the mean number of tumors in the mice treated with quercetin was 5 ± 0.92 (*n* = 10), which represents a 50% reduction in tumor number after quercetin treatment. There was also a significant difference in the mean tumor size between the AOM/DSS-treated mice and the quercetin+AOM/DSS cotreated mice (Figure 2(b)). In the mice, we also investigated for mucin-depleted foci and aberrant crypt foci, two preneoplastic lesions that are often used as biomarkers in colon carcinogenesis. As shown in Figures 2(c) and 2(d), the AOM/DSS-treated mice showed a significant elevation in the number of mucin-depleted foci and aberrant crypt foci when compared to the control group, while the quercetin+AOM/DSS cotreated group showed decreased formations of mucin-depleted foci and aberrant crypt foci. These results indicate that quercetin effectively inhibits tumor growth in vivo. Recent studies have shown that inflammation is associated with the development and malignant progression of most cancers. Thus, we also investigated the incidences of inflammation in these groups. The results showed that the AOM/DSS-treated mice had a higher inflammatory index, while the quercetin+AOM/DSS cotreated group showed a decreased inflammatory index (Figure 4).

### 3.4. Quercetin Reduces Oxidative Stress in AOM/DSS-Treated Mice

**Figure 5:**
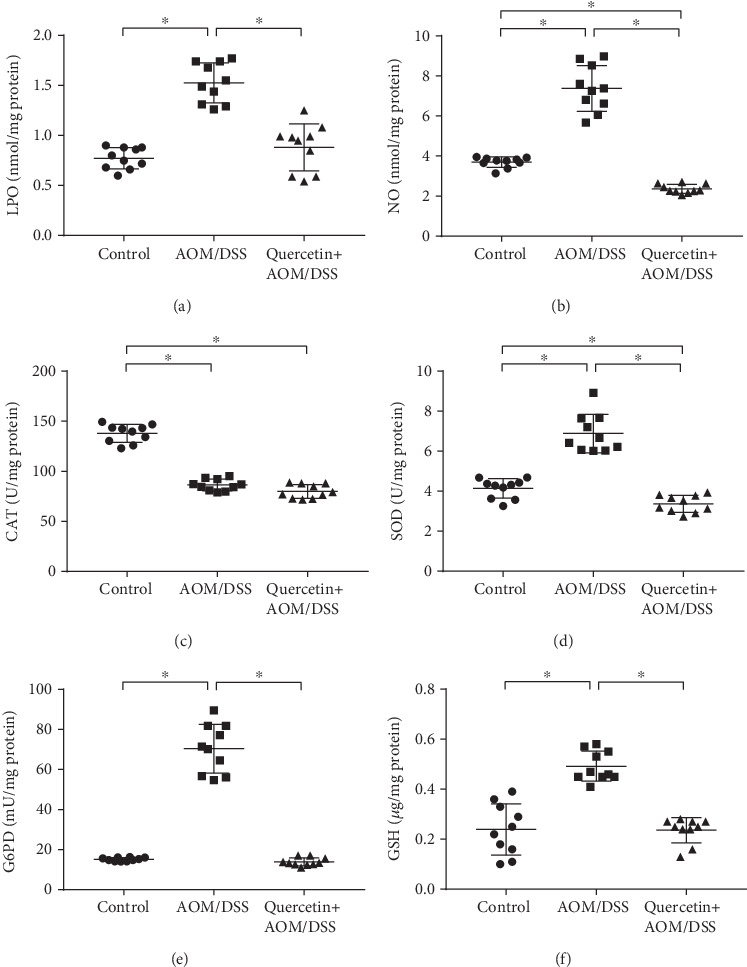
Quercetin reduces oxidative stress in AOM/DSS-treated mice. (a) Quantification of the LPO expression level in control and experimental mice colon tissues. (b) Quantification of the NO expression level in control and experimental mice colon tissues. (c) Quantification of the CAT expression level in control and experimental mice colon tissues. (d) Quantification of the SOD expression level in control and experimental mice colon tissues. (e) Quantification of the G6PD expression level in control and experimental mice colon tissues. (f) Quantification of the GSH expression level in control and experimental mice colon tissues. Data are expressed as mean values ± SD (*n* = 10/group). ^∗^*P* < 0.05, Student's *t*-test.

Wide-ranging studies during the last 30 years have revealed the mechanisms by which oxidative stress causes chronic inflammation, which in turn can lead to several chronic diseases, including cancer. For this reason, we sought to eliminate the expression of oxidative stress markers in the mice used in this study. A significant (*P* < 0.05) increase in the levels of enzymatic antioxidants (LPO, NO, and SOD) and glutathione-metabolizing enzymes (G6PD and GSH) was observed in the AOM/DSS-treated mouse when compared to the control group (Figures 5(a)–5(f)). Surprisingly, the administration of quercetin along with AOM/DSS significantly (*P* < 0.05) reduced the levels of LPO, NO, SOD, G6PD, and GSH back to the levels of the control group (Figures 5(a)–5(f)). However, cotreatment of the mice with AOM/DSS and quercetin did not restore the expression of CAT (Figure 5(c)). Nevertheless, this data demonstrated that quercetin could reduce oxidative stress in the AOM/DSS-treated mice, suggesting the underlying mechanism of its antitumor effect.

### 3.5. Quercetin Restores Cell Proliferative Activity in AOM/DSS-Treated Mice

**Figure 6:**
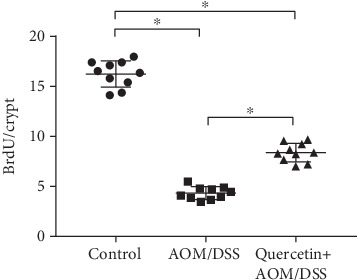
Quercetin restores cell proliferative activity in AOM/DSS-treated mice. Quantification of BrdU staining. BrdU cells per crypt were elevated; a total of 20 crypts per mouse; *n* = 10 per group. ∗*P* < 0.05.

Next, we investigated the proliferative activity of colon cells in vivo. The number of BrdU-positive cells per crypt was used to indicate the proliferative rate of the colon cells. As shown in Figure 6, AOM/DSS treatment dramatically decreased the number of BrdU-positive cells in the mice when compared with that in the control group. When compared with the AOM/DSS group, the quercetin+AOM/DSS cotreated group showed significant AOM/DSS-mediated decreases in BrdU-positive cells (Figure 6). This data showed the protective effect of quercetin against AOM/DSS.

### 3.6. Quercetin Alters Metabolite Profiling

**Figure 7:**
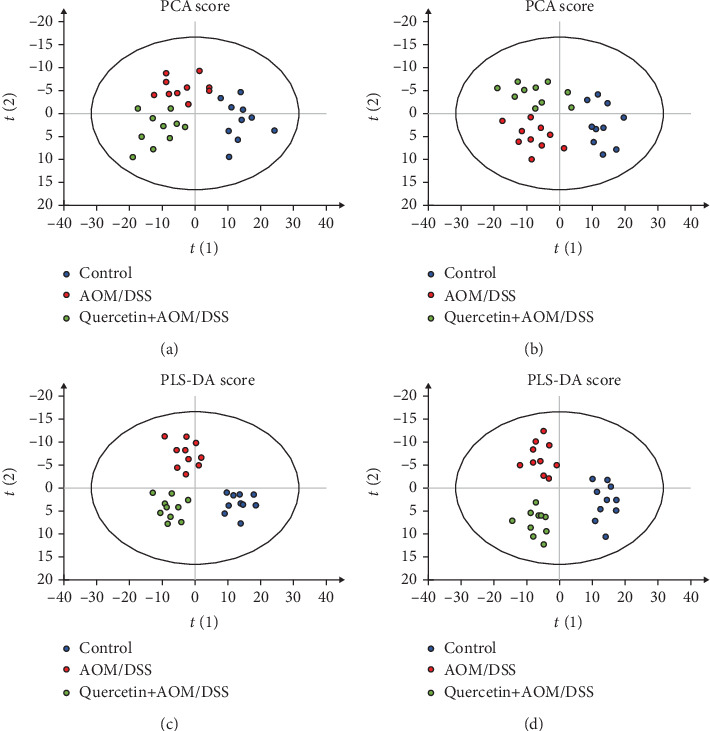
Quercetin alters serum metabolites in AOM/DSS-treated mice. (a) PCA score from serum in positive mode. (b) PCA score from serum in negative mode. (c) PLS-DA score from serum in positive mode. (d) PLS-DA score from serum in negative mode. *n* = 10 per group.

We also investigated the metabolism variations and potential effects of quercetin in the serum. As shown in Figure 7, PCA and PLS-DA were performed in positive and negative ion mode. An approximate separation was found in the control, AOM/DSS, and quercetin+AOM/DSS groups (Figures 7(a) and 7(b)). The PLS-DA also showed satisfactory classification (Figures 7(c) and 7(d)). As shown in Table 1, ten metabolites in the serum were selected from the results of the *t*-test (*P* < 0.05), fold change (<0.6–>1.5), and variable importance in the projection (VIP) (>1). Four of the metabolites, including 2-hydroxybutyrate, 2-aminobutyrate, 2-oxobutyrate, and endocannabinoids, were significantly higher in the AOM/DSS group than in the control group (*P* < 0.05). In addition, five of the metabolites, including gentian violet, indole-3-methyl acetate, N-acetyl-5-hydroxytryptamine, indoxyl sulfate, and indoxyl, were significantly decreased in the AOM/DSS group when compared to the control group (*P* < 0.05). In contrast, the levels of 2-hydroxybutyrate, 2-aminobutyrate, 2-oxobutyrate, endocannabinoids, and sphinganine were significantly lower in the quercetin+AOM/DSS group when compared to the AOM/DSS group (*P* < 0.05), while the levels of gentian violet, N-acetyl-5-hydroxytryptamine, indoxyl sulfate, and indoxyl were significantly higher in the quercetin+AOM/DSS group than in the AOM/DSS group (*P* < 0.05).

## 4. Discussion

In the present study, we demonstrated that quercetin reduced colorectal tumor proliferation in AOM/DSS-treated mice, prevented colonic inflammation by changing the lymphocyte counts, decreased oxidative stress in the colon, and modulated the expression of oxidative stress proteins.

During inflammation, leukocytes and other immunocytes are recruited to the site of damage, which results in a “respiratory burst” caused by an increased uptake of oxygen, in turn leading to an elevated release and accumulation of ROS in the area of damage [23, 24]. Following a long-lasting inflammatory stimulus, the initiation of carcinogenesis mediated by ROS could be direct or indirect, such as through signaling pathways activated or associated with ROS [25, 26]. In our experiment, a colon cancer model was established by feeding mice AOM/DSS, resulting in significant increases in inflammatory and ROS markers. Quercetin was reported as a long-lasting anti-inflammatory compound that possesses strong anti-inflammatory and antioxidation capacities in vitro [22]. In our study, we showed its striking anti-inflammatory and antioxidation effects in vivo. Furthermore, quercetin also exhibited antitumor proliferation and mucin-depleted foci and aberrant crypt foci inhibition effects. Since inflammation and ROS are strongly linked with cancer development, we concluded that the main anticancer effect of quercetin may come from its antioxidation function. However, how quercetin regulates ROS remains unknown.

The activation of immune signaling pathways by chemical or bacterial stimuli results in a loss of homeostasis in immunity that drives a proneoplastic inflammatory environment [27]. The inflammatory characteristics involved in colorectal carcinogenesis include inflammasome activation and activation of the NF-*κ*B pathway, both of which can occur by changes in the mutational landscape or in response to either chemical stimuli or cytokines [28, 29]. One of the key innate factors of the inflammatory response that contributes to CRC progression is ROS, which serves as a genotoxic compound driving the accumulation of mutations within proliferating epithelial cells [30, 31]. Our data showed that treatment with quercetin in mice receiving AOM/DSS restored the RBC, WBC, lymphocyte, and eosinophil counts, indicating the protective effect of quercetin on the hemopoietic system and its ability as an anti-inflammation compound. This data is consistent with previous findings [22].

Because the mortality rate for CRC in men and women ranks 4th and 3rd among cancer-related deaths, respectively, it is critical to find a novel screening method for identifying CRC in a timely manner [32]. Recently, metabolomics was developed as a powerful tool to detect potential biomarkers for cancer. A few studies have also used metabolomics to analyze the different metabolites in CRC. In keeping with previous reports [32–34], our study showed that increased levels of 2-hydroxybutyrate, 2-aminobutyrate, and 2-oxobutyrate and decreased levels of gentian violet, indole-3-methyl acetate, N-acetyl-5-hydroxytryptamine, indoxyl sulfate, and indoxyl were found in AOM/DSS-treated mice. This finding is significant because 2-hydroxybutyrate is usually considered to be a marker of GSH status [35], while 2-aminobutyrate is a precursor to synthesize ophthalmate [36], an indicator of GSH metabolism through the activation of *γ*-glutamyl cysteine synthetase. Thus, the elevation of 2-aminobutyrate and 2-hydroxybutyrate implied a greater amount of oxidative stress in the AOM/DSS-treated mice.

In conclusion, the findings of our present research uncovered a significant role for ROS and inflammation in the pathogenesis of AOM/DSS-induced colon toxicity and the initiation of colon tumors. Furthermore, our study demonstrated that quercetin has a positive beneficial effect against colon cancer progression in the AOM/DSS-induced mice colon cancer model.

## Figures and Tables

**Table 1:** Metabolite profiling in the serum from the control, AOM/DSS, and quercetin+AOM/DSS groups.

	Control vs. AOM/DSS			Control vs. quercetin+AOM/DSS			AOM/DSS vs. quercetin+AOM/DSS		
	VIP	*t*-test	Trend	VIP	*t*-test	Trend	VIP	*t*-test	Trend
2-Hydroxybutyrate	1.4378	0.0011	↑	1.6286	0.0012	↑	2.2176	00028	↓
2-Aminobutyrate	1.2419	0.0025	↑	1.3547	0.0018	↑	1.8765	0.0078	↓
2-Oxobutyrate	1.3043	0.0176	↑	1.2365	0.0043	↑	1.7458	0.0160	↓
Endocannabinoid	1.6786	0.0148	↑	1.4298	0.0035	↑	1.6385	0.0087	↓
Sphinganine	—	—	—	—	—	—	1.6643	0.0492	↓
Gentian violet	1.3345	0.0049	↓	—	—	—	1.4250	0.0087	↑
Indole-3-methyl acetate	1.4261	0.0250	↓	—	—	—	—	—	—
N-Acetyl-5-hydroxytryptamine	1.3276	0.0251	↓	—	—	—	1.3462	0.0132	↑
Indoxyl sulfate	1.3534	0.0142	↓	—	—	—	1.6241	0.0176	↑
Indoxyl	1.7664	0.0352	↓	1.4096	0.0230	↓	1.1295	0.0089	↑

Biomarkers were selected according to the *t*-test (*P* < 0.05), fold change (<0.6–>1.5), and VIP (>1) results.

## Data Availability

All the data in this study are available upon request through the correspondence author.
